# Wound healing approach based on excretory-secretory product and lysate of liver flukes

**DOI:** 10.1038/s41598-022-26275-y

**Published:** 2022-12-14

**Authors:** Anna V. Kovner, Alena A. Tarasenko, Oxana Zaparina, Olga V. Tikhonova, Maria Y. Pakharukova, Viatcheslav A. Mordvinov

**Affiliations:** 1 Institute of Cytology and Genetics, Siberian Branch of Russian Academy of Sciences (ICG SB RAS), 10 Lavrentiev Ave., Novosibirsk, 630090 Russia; 2 Department of Natural Sciences, Novosibirsk State University, 2 Pirogova Str., Novosibirsk, 630090 Russia; 3 Institute of Biomedical Chemistry (IBMC) RU, Pogodinskaya 10, Moscow, 119121 Russia

**Keywords:** Molecular biology, Skin diseases

## Abstract

Exogenous bioactive peptides are considered promising for the wound healing therapy in humans. In this regard, parasitic trematodes proteins may potentially become a new perspective agents. Foodborne trematode *Opisthorchis felineus* is widespread in Europe and has the ability to stimulate proliferation of bile duct epithelium. In this study, we investigated skin wound healing potential of *O. felineus* proteins in mouse model. C57Bl/6 mice were inflicted with superficial wounds with 8 mm diameter. Experimental groups included several non-specific controls and specific treatment groups (excretory-secretory product and lysate). After 10 days of the experiment, the percentage of wound healing in the specific treatment groups significantly exceeded the control values. We also found that wound treatment with excretory-secretory product and worm lysate resulted in: (i) inflammation reducing, (ii) vascular response modulating, (iii) type 1 collagen deposition promoting dermal ECM remodeling. An additional proteomic analysis of excretory-secretory product and worm lysate samples was revealed 111 common proteins. The obtained data indicate a high wound-healing potential of liver fluke proteins and open prospects for further research as new therapeutic approaches.

## Introduction

Chronic non-healing wounds or abnormal scarring is a major public health problem worldwide. There are numerous causes of such pathological conditions: severe mechanical and burn injuries, obesity and physical inactivity, diseases of cardiovascular system and autoimmune diseases, including diabetes mellitus, as well as a number of other diseases^1–3^. Normally, skin regeneration is a natural homeostatic process that includes four successive stages: (1) hemostasis (platelet activation, clot formation to prevent blood loss and fibrin matrix formation, up to 48 h); (2) inflammation (immune response, activation of inflammatory cells, up to 96 h); (3) proliferation (modulated M2 to M1 polarization of macrophages, keratinocyte migration, angiogenesis activation, tissue granulation, epithelial folds and wet crust/eschar, up to 10–14 days); (4) remodeling (maturation of epidermis, overall reduction of wound area, regression of blood vessels, structuring of extracellular matrix, more than 10 days)^4–6^. In case of pathological no overgrowth, wounds do not undergo normal stages of healing, linger at stage of inflammation or proliferation and remain incurable despite intense therapeutic treatment^7,8^.

Promising therapeutic approaches to wound healing correction include various gels, growth factors, herbal preparations, natural polysaccharides (chitosan) and bioactive peptides of various origins^9–12^. Due to this, proteins of parasitic trematodes may potentially become novel perspective agents for wound healing, as well as treatment of autoimmune diseases^13–15^. Currently, helminth therapy is considered as an experimental method for treatment of certain autoimmune diseases: Crohn's disease, celiac disease, multiple sclerosis, asthma and etc*.*^16–18^. Given the role in a regulation of host’s immune response—bioactive and regulatory molecules of parasites may be considered a relevant therapeutic method. These regulatory molecules allow parasite to control host’s local immune response and exist in mammalian organism for a long time.

The foodborne liver trematode *Opisthorchis felineus is* one of the most well-known and harmful Opisthorchiidae parasites. This parasite is widespread in Eurasia, including Western Europe. Prevalence of human infection is highest in Russia, where up to 40,000 cases of *O. felineus* infection are diagnosed annually^19^. Disease caused by this parasite is characterized by a long course and may be asymptomatic for a long time. *O. felineus* is able to reduce acute inflammation caused by invasion and stimulate repair of damaged liver epidermis and tissue remodeling^20,21^. It becomes well documented that parasite-derived bioactive substances and regulatory molecules may actively improve wound healing in mammals^14^. In db/db mice model of diabetes mellitus, treatment with *Schistosoma japonicum* soluble egg antigen (SEA) showed a significant reduction in insulin resistance and increased wound healing^22^. An orthologue of granulin from human parasitic liver fluke *Opisthorchis viverrini*, known as Ov-GRN-1, induces angiogenesis and accelerates wound repair^14,23^. However, despite the proximity of *O. felineus* and *O. viverrini*, they have different carcinogenic potential: 3A and 1A, respectively^19^. Probably the difference in carcinogenicity can be associated with differences in wound healing potential. Thus, the aim of this study was to test *Opisthorchis felineus* excretory-secretory product and lysate proteins as wound healing agents on a murine model.

## Results

### Visual assessment of wound area

**Figure 1:**
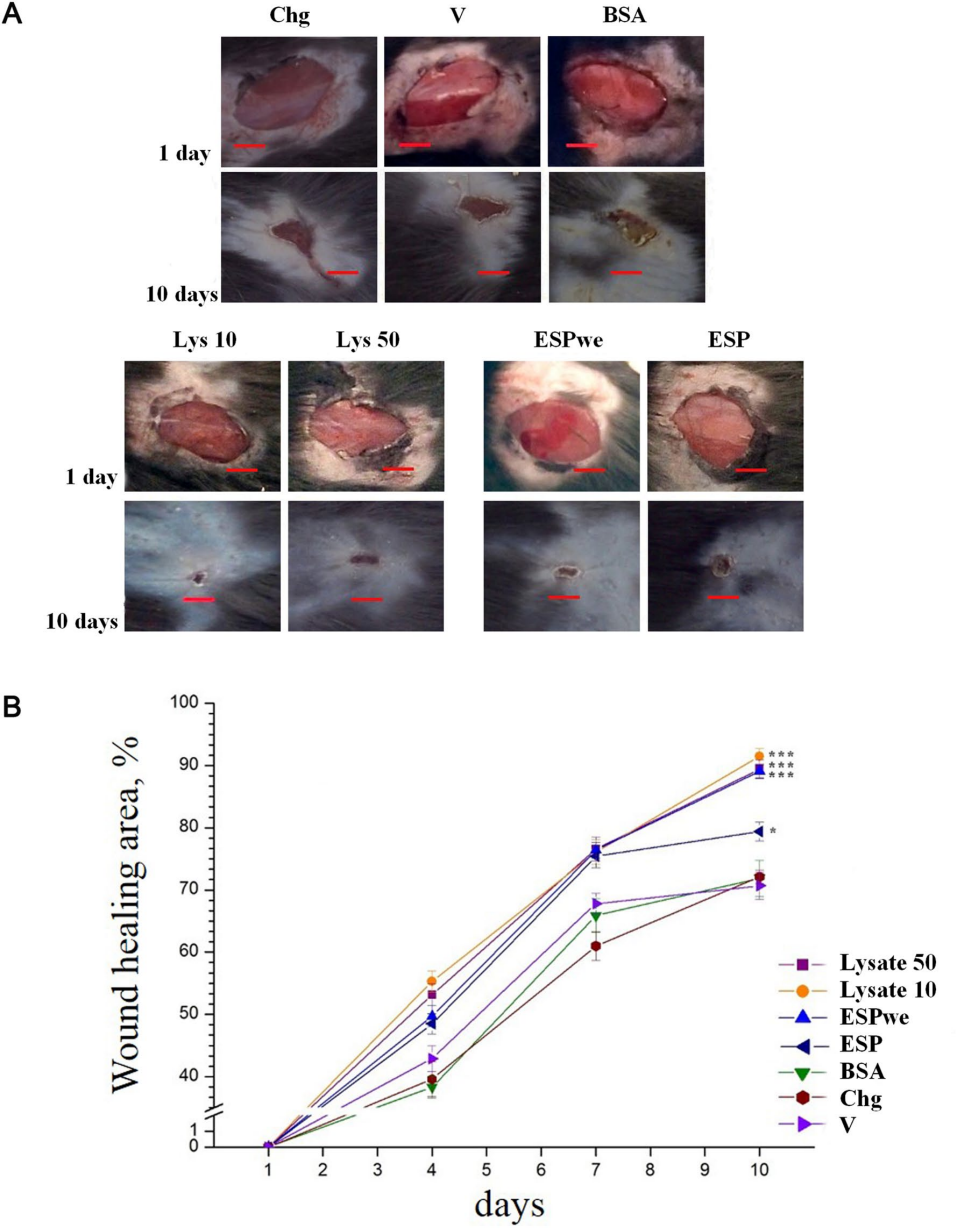
Visual scoring of wound healing. (**A**) Wound healing on days 1 and 10 after treatment. Wounded untreated, vehicle and nonspecific control and specific treatment groups (3 mm scale bar); (**B**) Wound healing rate chart. Data is presented for area percentage as mean ± SEM, *p < 0.05, ***p < 0.001 as compared to vehicle group.

After 10 days of the experiment (experiment scheme is shown in Fig. 7), there was an increase in wound healing compared to non-specific control groups in all groups of specific treatment (Fig. 1A). Visual assessment was used to quantify wound area and the reduction of the wound size. The area of wound healing was higher in all groups of specific treatment, significantly different from vehicle group. The highest percentage of wound healing was detected in Lys 10, Lys 50 and ESPwe groups (p < 0.000161, p < 0.000248, p < 0.000291 respectively) (Fig. 1A,B). When comparing two concentrations of worm lysate, it was found that their effect on wound closure is the quite similar. For this reason, further in the work, ESPwe and Lysate 10 μg (the lower of the two concentrations) were used as the two most effective groups.

### Stages of wound healing

Material sampling for detection of the stage of wound healing was carried out at the last two stages of wound healing—proliferation and/or remodeling (7 and 10 days after treatment). Semi-quantitative analysis of H&E stained slides showed that in vehicle, untreated and non-specific control groups on the 10th day of the experiment, all tissue samples taken from the wound area remained at the proliferation stage: the presence of a wet crust, epithelial ridges and significant infiltration areas (both inflammatory and granulation tissues). In all specific treatment groups, wound tissue samples were at the stage of remodeling: lack of wet crust, reduced infiltration of areas (mainly macrophage and granulation tissue). In the Lysate 10 µg and 50 µg groups, the initial processes of re-epithelialization (closing of epithelial ridges) were detected (Table 1).

**Table 1:** Semi-quantitative analysis of skin pathomorphological changes after injury.

	Chg		BSA		V		ESP		ESPwe		Lys10		Lys50	
	days													
	7	10	7	10	7	10	7	10	7	10	7	10	7	10
Wet crust	+	+	+	+	+	+	+	−	+	−	+	−	+	−
Epithelial ridge	+	+	+	+	+	+	+	+	+	+	+	−	+	−
Infiltrate	+++	++	+++	++	+++	+++	++	+	++	+	++	+	++	+

**Figure 2:**
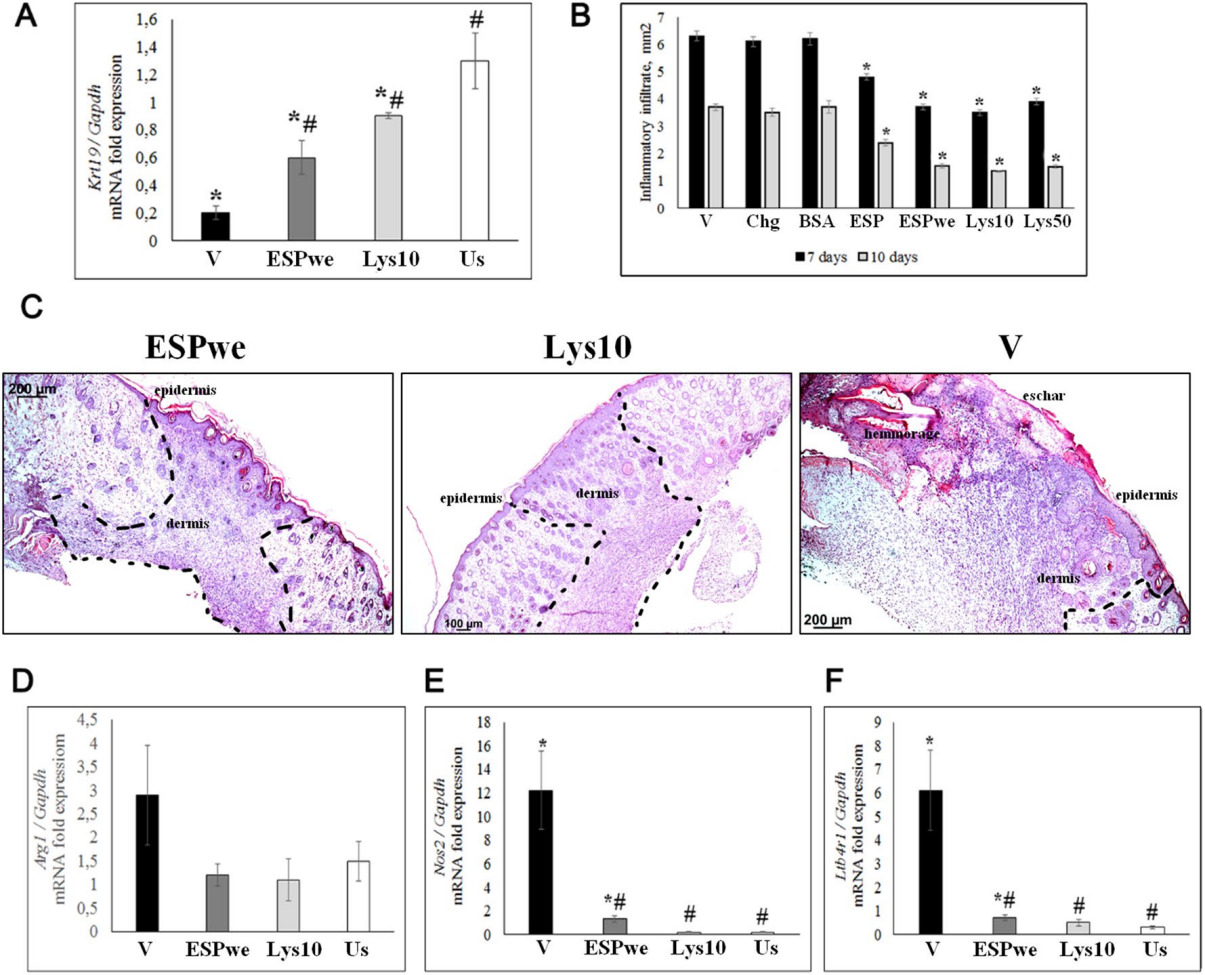
General wound healing evaluation. (**A**) Cytokeratin 19 (*Krt19*) gene expression normalized to *Gapdh* gene expression. *V* vehicle, *ESPwe* excretory-secretory product without endotoxin 10 µg, *Lys 10* Lysate 10 µg, *Us* unwounded healthy skin. Data are presented as mean ± SEM, *p ≤ 0.05 as compared to unwounded skin, ^#^p ≤ 0.05 as compared to a vehicle; (**B**) Infiltrate area, mm^2^. *V* vehicle, *Chg* chlorhexidine, *BSA* bovine serum albumin, *ESP* excretory secretory product 10 µg, *ESPwe* excretory secretory product without endotoxin 10 µg, *Lys 10* Lysate 10 µg, *Lys 50* Lysate 50 µg. Data are presented as mean ± SEM, *p < 0.05 as compared to a vehicle; (**C**) Wound area, hematoxylin and eosin staining, 10 days after treatment. The dotted line marked the wound area; (**D**) *Arg1* gene expression normalized to *Gapdh* gene expression; (**E**) *Nos2* gene expression normalized to *Gapdh* gene expression; (**F**) Leukotriene receptor B4 (*Lblt4l*) gene expression normalized to *Gapdh* gene expression; (**D**–**F**) Data are presented as mean ± SEM, *p ≤ 0.05 as compared to unwounded healthy skin, ^#^p ≤ 0.05 as compared to a vehicle.

Level of *Krt19* gene expression (molecular marker of skin epidermis) may indirectly indicate the degree of wound epithelialization. The expression of this gene in the Lysate 10 µg and ESP was significantly different from vehicle and unwounded healthy skin groups (Fig. 2A). Lysate 10 µg and ESPwe groups were taken into analysis as the most effective of specific treatment groups, respectively. Morphometric analysis of the infiltrate area (mm^2^) revealed a significant decrease in all groups under specific treatment compared to vehicle group. The infiltrate was dominated by lymphocytic-macrophage cells. In the ESPwe and Lysate 10 µg groups, the smallest area of infiltrative changes was detected (Fig. 2B,C). In addition, to study the effect of *O. felineus* ESP and lysate proteins on a change in the stages of wound healing and the degree of inflammation, the expression of molecular markers for M1/M2 macrophages (*Nos2*/*Arg1*) and the inflammatory mediator B4 1 leukotriene receptor was analyzed. The expression of all selected genes in specific treatment groups was comparable to unwounded healthy skin (Fig. 2D–F).

**Figure 3:**
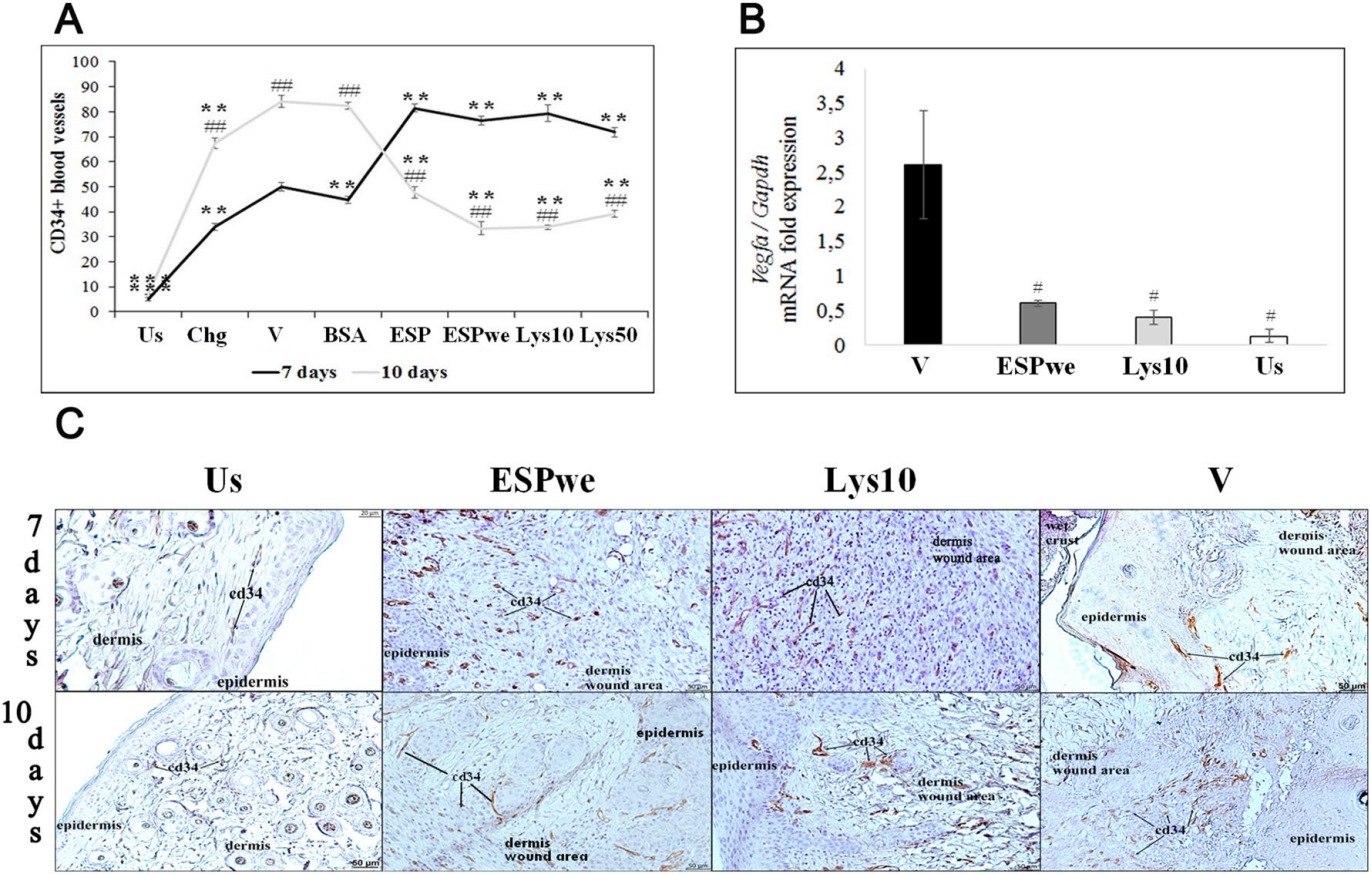
Neoangiogenesis study. In all groups of specific treatment, a significant decrease of newly formed vessels was detected. (**A**) Number of CD34+ blood vessels per a total wound area. *Us* unwounded healthy skin, *V* vehicle, *Chg* chlorhexidine, *BSA* bovine serum albumin, *ESP* excretory secretory product, *ESPwe* excretory secretory product without endotoxin, *Lys 10* Lysate 10 µg, *Lys 50* Lysate 50 µg. Data are presented as mean ± SEM, **p < 0.01 as compared to a vehicle, ^##^p < 0.01 as compared to previous study period.; (**B**) *Vegfa* gene expression normalized to *Gapdh* gene expression. *V* vehicle, *ESPwe* excretory secretory product without endotoxin, *Lys 10* Lysate 10 µg, *Us* unwounded healthy skin. Data are presented as mean ± SEM, *p ≤ 0.05 as compared to unwounded skin, ^#^p ≤ 0.05 as compared to a vehicle; (**C**) CD34 + staining for newly formed vessels.

Immunohistochemical analysis of neoangiogenesis (CD34 marker of newly formed vessels) showed that in the groups of specific treatment, this parameter significantly decreased from day 7 to 10 of the experiment. This may indicate a successful change in the phases of wound healing from proliferation to remodeling. In untreated, vehicle and nonspecific control groups, the number of newly formed vessels increased by day 10 of the experiment (Fig. 3A,C). Vascular endothelial growth factor (Vegfα) is a key dynamic molecule of angiogenesis. On day 10 of the experiment, in the groups of specific treatment, the expression of the *Vegfa* gene is higher, compared to intact unwounded healthy skin, but lower than in the vehicle group (Fig. 3B). Thus, *O. felineus* ESP and lysate proteins are able to accelerate the change of wound healing phases from proliferation to remodeling due to the regression of newly formed vessels.

**Figure 4:**
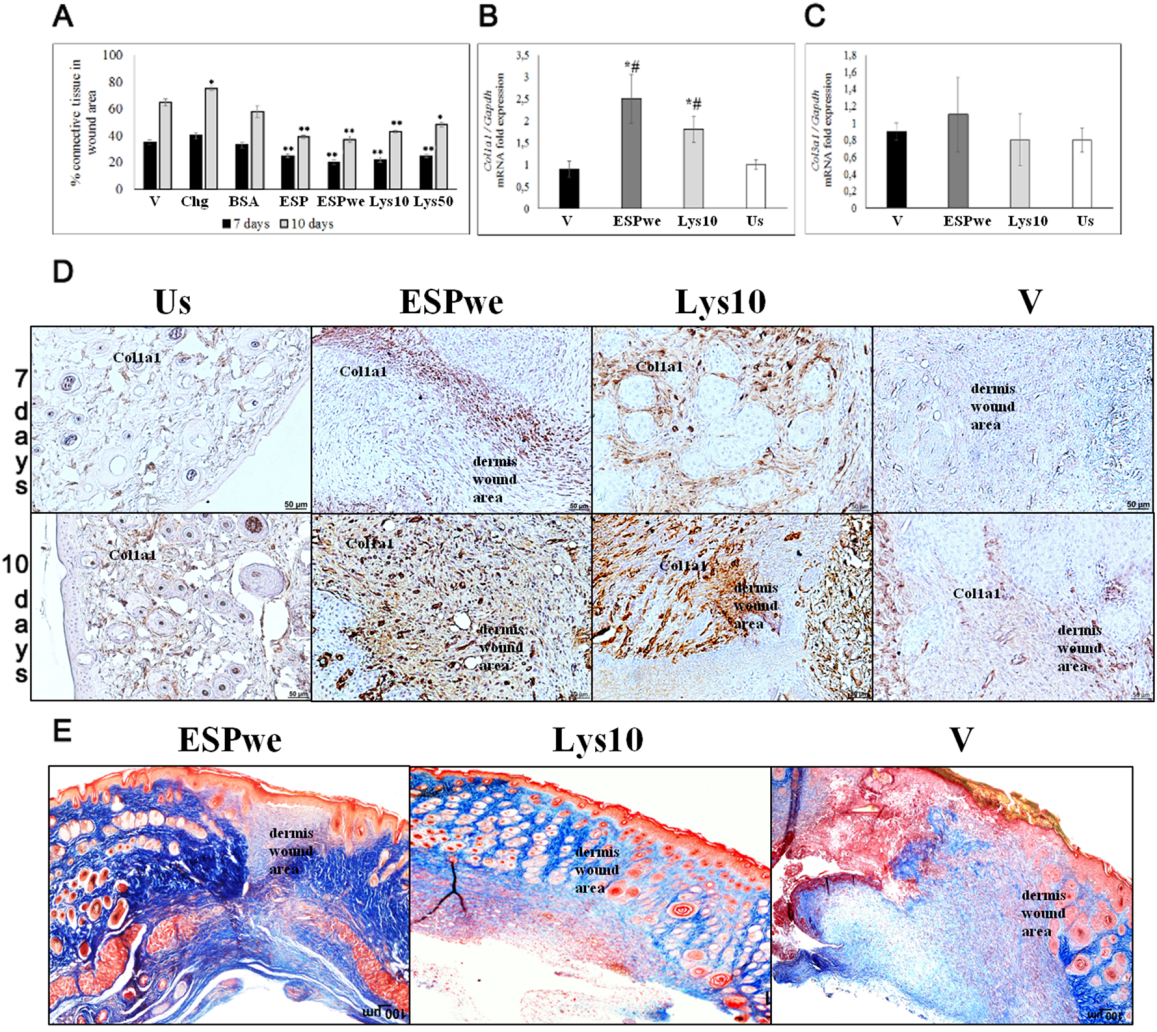
Skin extracellular matrix study. In all groups of specific treatment, a significant decrease of connective tissue area was detected. (**A**) Percentage of connective tissue in a wound area. *V* vehicle, *Chg* chlorhexidine, *BSA* bovine serum albumin, *ESP* excretory secretory product, *ESPwe* excretory secretory product without endotoxin, *Lys 10* Lysate 10 µg, *Lys 50* Lysate 50 µg. Data is presented as mean ± SEM, *p < 0.05; **p < 0.01 as compared to a vehicle; (**B**,**C**) *Col1a1*, *Col3a1* gene expression normalized to *Gapdh* gene expression. *V* vehicle, *ESPwe* excretory secretory product without endotoxin, *Lys 10* Lysate 10 µg, *Us* unwounded healthy skin. Data is presented as mean ± SEM, *p ≤ 0.05 as compared to unwounded healthy skin group, ^#^p ≤ 0.05 as compared to a vehicle group; (**D**) Col1a1 + fibers, visual increasing in specific treatment groups; (**E**) Connective tissues in a wound area, Mallory staining.

An assessment of percentage of connective tissue in a wound area showed that in all groups this parameter increased between days 7 to 10 of the experiment. However, in tissue samples from specific treatment groups, this percentage was lower than in vehicle group (Fig. 4A,E). *O. felineus* ESP and lysate proteins reduce amount of connective tissue in a wound area, which may indicate an effective skin regeneration. In specific treatment groups (ESP, ESPwe, Lys 10, Lys 50), physiologically normal arrangement of epidermis and dermis was detected in tissue samples. Study of gene expression of two main types of skin collagen—collagen 1, 3—showed that in ESPwe and Lysate 10 µg groups, *Col1a1* gene expression was differ from the level of expression in vehicle and unwounded healthy skin. Expression level of *Col3a1* gene in ESPwe and Lysate 10 µg groups did not differ significantly from both control group (Fig. 4B,C). A visual increase in deposition of Col1a1+ collagen in ECM in wound area on day 10 of the experiment should also be noted (Fig. 4D).

**Figure 5:**
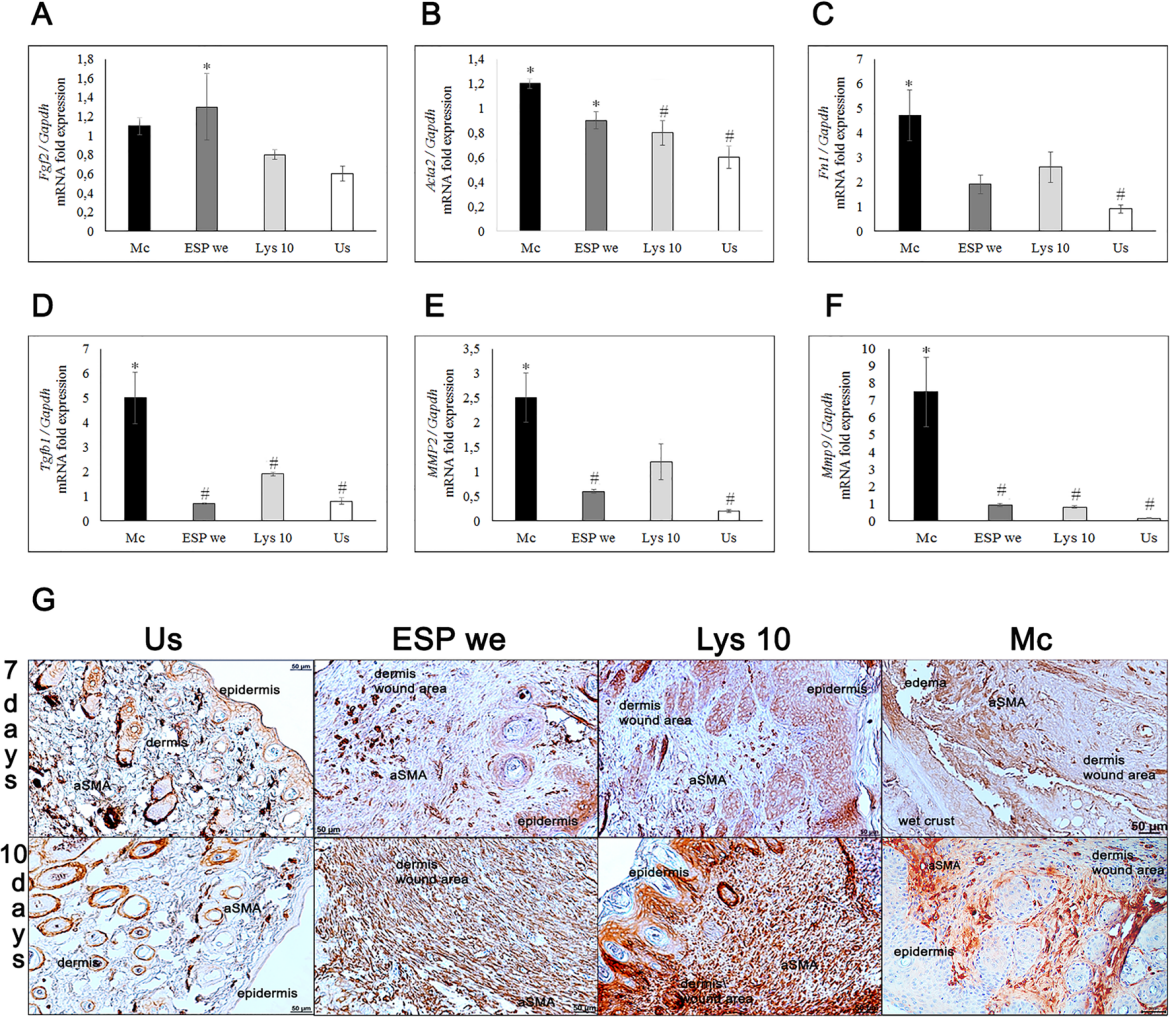
Skin extracellular matrix study. Specific treatment (ESPwe and Lysate 10 µg) significantly reduced expression of genes responsible for ECM remodeling. (**A**–**F**) *Fgf2*, *Acta2*, *Fn1*, *Tgfb1*, *Mmp2*, *Mmp9* genes were normalized to average *Gapdh* expression. Data is presented as mean ± SEM, *p ≤ 0.05, **p < 0.01 as compared to unwounded healthy skin group, ^#^p ≤ 0.05 as compared to a vehicle group; (g) α-SMA positive cells and tissue fibers in a wound area and in an un-wounded healthy skin.

By day 10 of the experiment, expression of *Fgf2* (fibroblast growth factor) gene in ESPwe and Lysate 10 µg groups did not significantly differ from vehicle group. However, expression of this gene in ESPwe group was significantly higher than the level of expression in unwounded healthy skin (Fig. 5A). This was accompanied by a significant activation of expression of α-smooth muscle actin (*Acta2*) gene and synthesis of a significant amount of collagen I and III, as well as other ECM proteins. Presence of myofibroblasts at early stages of wound healing leads to a reduction in size of a wound, but their excessive number can lead to improper collagen deposition and, as a result, appearance of hypertrophic scars. In addition to collagen deposition in the wound area, a decrease in *Acta2* gene expression were also detected in ESP group, but not in Lysate 10 µg group (Fig. 5B). However, αSMA visual increase was detected in both specific treatment groups (Fig. 5G). Another important ECM structural protein is fibronectin. Fibronectin fibrils are among first ECM proteins to assemble during wound healing. By day 10 in Lysate 10 µg and ESPwe groups *Fn1* gene expression remains similar to unwounded healthy skin (Fig. 5C). In both treatment groups, expression of genes associated with the state of remodeling of extracellular matrix, *Tgfb1* and matrix metalloproteinases (*Mmp2*, *Mmp9*) was closer to unwounded healthy skin in comparison to vehicle group (Fig. 5D–F). Thus, *O. felineus* ESP and lysate proteins are able to stimulate collagen formation and ECM remodeling in a wound area, which may indicate high-quality skin regeneration.

### Proteomic analysis

**Figure 6:**
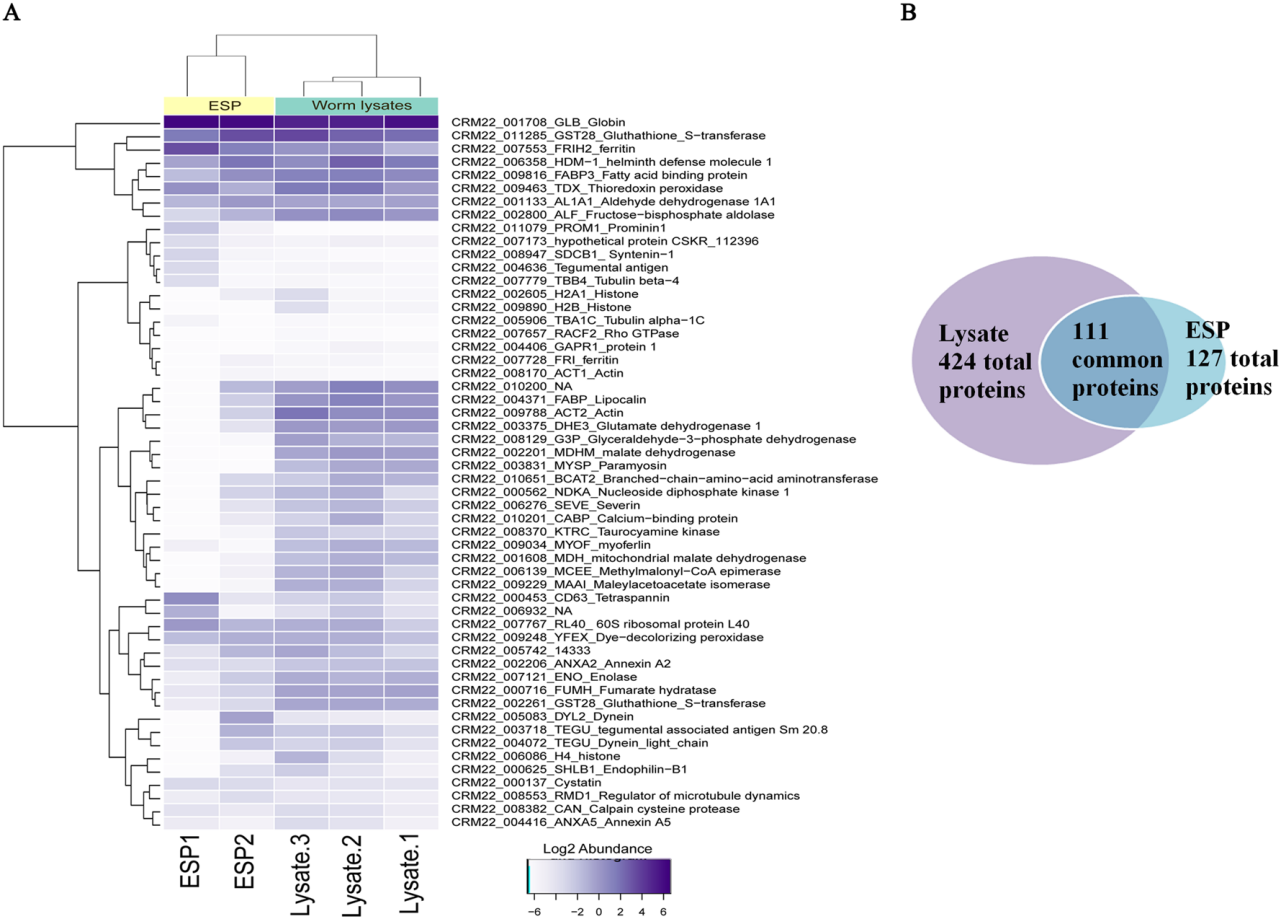
Proteomic analysis of ESP and lysate samples. (**A**) Heat map of major proteins presented as log2 percent abundance; (**B**) Common proteins for ESP and lysate samples.

Proteomic analysis of ESP and lysate samples identified more than 127 and 424 proteins, respectively (filtered by abundance > 0.01%) (Supplementary material, supplementary 7 “proteomic analysis (ESP and Lysate samples)”). The mass spectrometry proteomics data have been deposited to the ProteomeXchange Consortium via the PRIDE^24^ partner repository with the dataset identifier PXD037991. Function of many of them is unknown. Major proteins that make up a bulk of ESP product are 9 proteins (globin, glutathione-S-transferase, thioredoxin peroxidase, tetraspanin CD63, ribosomal protein L40, helminth defense molecule 1, ferritin, fatty-acid-binding protein, aldehyde dehydrogenase 1A1), rest are represented by less than 1%. Lysate samples contain almost same major proteins, except for tetraspanin CD63, concentration of which drops by more than 10 times and becomes about 0.1%. In addition, major fraction (more than 1%) of lysate samples also includes metabolic enzymes such as glutamate dehydrogenase 1, fructose-bisphosphate aldolase. Thus, major fractions can be determined, which include:

1. heme-associated proteins (globin (73.1% ESP samples, 44.5% Lysate samples), ferritin (6.6% ESP samples, 1.1% Lysate samples), DyP-type peroxidase (0.4% ESP samples, 0.4% Lysate samples);
2. proteins with a detoxification function (GST28 (6.9% ESP samples, 7.9% Lysate samples); ALT1A1 (0.7% ESP samples, 0.7% Lysate samples); GST28_1 (0.1% ESP samples, 0.62% Lysate samples));
3. proteins with antioxidant activity (TPX (0.9% ESP samples, 2.5% Lysate samples));
4. proteins modulating immune response (helminth defense molecule 1 HDM1 (2.2% ESP samples, 3.8% Lysate samples), FABP3 (0.9% ESP samples, 2.3% Lysate samples); Lipocalin (0.1% ESP samples, 1.6% Lysate samples));
5. proteins associated with calcium metabolism (Anxa2 (0.07% ESP samples, 0.15% Lysate samples));
6. interaction with fibrinolytic system (Enolase-C (0.1% ESP samples, 0.4% Lysate samples));
7. host-parasite interactions (ALF (0.3% ESP samples, 1.4% Lysate samples));
8. exosome-containing proteins (Tetraspannin CD63 (0.83% ESP samples, 0.12% Lysate samples));
9. protein degradation (Ubiquitin (0.7% ESP samples, 0.4% Lysate samples)) (Fig. 6A,B). Further in-depth analysis is likely to highlight major potential parasitic candidate proteins or exosomes that promote wound healing.

In addition, we carried out IHC staining for Opisthorchis felineus major proteins (GST and Tpx)^25,26^. TPX and GST were detected in epidermis and dermis cells in wound area on the 7th day of treatment (supplementary material 2). This demonstrates the ability of parasite proteins to penetrate into the wound area.

## Discussion

For the first time we have demonstrated that proteins of trematode *Opisthorchis felineus* possess wound healing capabilities. Groups with the most effective treatment included ESPwe and Lysate in different concentration. Also, using complex broad morphological approach, for the first time we have shown that *O. felineus* proteins promote: (i) acceleration of wound healing timeline; (ii) angiogenesis activation; (iii) decreased inflammation and (iv) ECM remodeling in vivo. We also used the model of multiple application of proteins for the first time for maximum effect, and approximation to treatment regimens in humans^12^.

Neoangiogenesis—formation of new blood vessels—plays a central role in wound healing processes, being induced by factors such as vascular endothelial growth factor (Vegfa) and fibroblast growth factor (Fgf2)^27^. During a normal course of wound healing processes, there is a decrease in the level of pro-inflammatory cytokines, and neovascularization is suppressed at the stage of remodeling. On one hand, activation of angiogenesis during wound healing improves tissue trophism, decreases the level of hypoxia and the migration/proliferation of cells^28,29^. On the other hand, inadequate activation of neoangiogenesis leads to hypertrophic scar formation^30^. ES protein, secreted granulin-like growth factor termed Ov-GRN-1 *O. viverrini*, have previously been shown to accelerate angiogenesis and wound healing in mice in vitro^31^. *S. haematobium* egg-secreted infiltrin protein has also been shown to be sufficient to induce angiogenesis in bladder^32^. In our study, for the first time the effect of parasite proteins on angiogenesis in the wound healing dynamics was demonstrated in vivo: application of samples from specific treatment groups (ESP, ESPwe, Lysate 10 µg, Lysate 50 µg) initially promotes an increase and then a decrease of newly formed CD34+ blood vessels. Expression levels of *Vegfa* and *Fgf2* genes on day 10 of experiment were also similar to expression levels in unwounded healthy skin. This may indicate canonical course of normal wound healing and normal phase change from proliferation to remodeling^33^.

Chronic helminth infection affects more than one billion people worldwide. They may suffer from complications caused by helminthiasis, but rarely develop allergic or chronic autoimmune diseases^34^. From an immunological point of view, this may be due to two main processes. First, helminthic invasion is accompanied by a strong Th2 response that suppresses acute inflammatory Th1 response. Second, chronic parasite infection can generate a pool of regulatory T (Treg) cells that secrete transforming growth factor TGFβ and IL-10. These cytokines not only regulate aggressive acute Th1 response, but also control enhanced Th2 response that contributes to the manifestation of chronic allergic diseases^34,35^. In addition to that, we have also previously shown that, three most numerous groups of differentially expressed genes in livers of *O. felineus*-infected hamsters were ECM-Receptor interaction, TGFβ signaling pathway and cell adhesion molecules^36^. Probably, activation of these groups of genes can also contribute to successful skin wound healing in vivo.

Successful change of stages from proliferation to remodeling is evidenced by a change of macrophage phenotype from M1 (pro-inflammatory) to M2 (reparative). It is necessary for fibroblast activation, which in turn stimulates keratinocyte proliferation and migration^37^. Parasitic nematodes are known to be able to activate M2 macrophage response in vivo and in vitro^38–40^. In our experiments, in specific treatment groups, a decrease in inflammatory infiltrated area of the wound was detected, as well as normal expression levels of *Nos2* (M1-macrophages) and *Arg1* (M2-macrophages) genes, comparable to unwounded healthy skin. Leukotriene B4 receptor 1 being an inflammatory mediator, stimulates the Th1 immune response^41^. On day 10, expression level of *Ltb4r1* gene in groups of specific treatment was comparable to unwounded healthy skin, also indicating canonical wound healing.

An important aspect of wound healing is high-quality regeneration, which does not lead to scarring of the skin. It is likely that scarring occurs due to excessive collagen deposition and excess α-SMA+ myofibroblasts during the proliferation phase of wound healing^42^. In unwounded healthy skin, collagen types I and III makes up the majority of dermal fibers (up to 85% and 11%, respectively). Collagen is one of the central players in wound healing processes. It participates in: (i) matrix formation for inflammatory cells; (ii) interaction with platelets; (iii) keratinocyte migration along temporary extracellular matrix; (iv) effective fibroblast differentiation^43^. Collagen is currently used in several commercially available skin wound healing products^44^. We have shown that in groups of specific treatment there is a visible increase in the number of Col1a1 positive fibrils in wound area. Expression of *Col1a1* gene in specific treatment groups was significantly higher than in unwounded healthy skin. This was accompanied by successful re-epithelialization processes—*Krt19* gene expression was comparable to unwounded healthy skin. In contrast to this, we did not find any significant changes in *Col3a1* gene expressions. Probably, all this is associated with the temporal dynamics of wound healing: type III collagen is gradually degraded, and type I collagen is generated at the same time^45^. *O. viverini* granulin has previously been shown to promote cholangiocyte proliferation in vitro^15^. In our in vivo experiment, we detected the ability of *O. felineus* proteins to stimulate the proliferation of keratinocytes.

Another important ECM protein is fibronectin, which modulates secretion of TGFβ-1 by fibroblasts and, as a result, the effectiveness of the synthesis of type 1 collagen^46^. Fn1 gene expression in both specific treatment groups did not differ from unwounded healthy skin. It is interesting to note that the expression of *Fn1* gene was the highest in the vehicle group. This may be associated with active processes of tissue remodeling and fibroblast stabilization^43^. Fibronectin 1 is a key player in coordinating the correct ECM formation, especially in the early stages of wound healing. In addition to that, Fn matrix impacts tissue organization by contributing to the assembly of other ECM proteins, including thrombospondin-1 and microfibrils^47^.

TGF-β1 is one of the key growth factors involved in wound healing processes, which promotes: (i) monocyte to macrophage differentiation, initiating granulation tissue development and releasing various pro-inflammatory cytokines and growth factors; (ii) differentiation of fibroblasts to myofibroblasts; (iii) in low levels—angiogenesis by increasing proliferation of endothelial cells and Vegfα expression, in high levels—hinders angiogenesis^43,48,49^. On day 10 after treatment, the expression level of *Tgfb1* gene in specific treatment groups was comparable to unwounded healthy skin.

Matrix metalloproteinases are necessary for effective regulation and completion of wound healing processes, as they cleave a wide range of extracellular proteins. Elevated MMP2/9 levels are associated with pro-inflammatory states and stimulated angiogenesis^50,51^. On day 10 of the experiment, expression of both *Mmp2* and *Mmp9* genes in specific treatment groups was comparable to unwounded healthy skin group, which may reflect positive dynamics of extracellular matrix remodeling in wound area. Therefore, we are first to demonstrate the ability of *O. felineus* proteins to stimulate wound healing.

Proteomic analysis of *O. felineus* ESP and lysate samples for the first time identified a major fraction of proteins. Among them, one can find “CRM22_006358_Helminth defense molecule”, a homologue of antimicrobial protein cathelicidin, previously described in other trematodes *Fasciola hepatica*^52,53^. Antimicrobial peptides, also known as endogenous host defense peptides, are peptides widely distributed in bacteria, plants, insects, amphibians, reptiles and mammals. Antimicrobial peptides are known to play an important role in innate immune response. Activation of innate immune system leads to antimicrobial peptides production, including by skin epidermis keratinocytes^54^. Potential exogenous stimulation of *O. felineus* helminth defense molecule 1 keratinocytes can serve as a nonspecific trigger of wound healing. Also, another protein that possibly modulates immune response is FABP—fatty acid binding protein (“CRM22_009816_FABP3”). It is known that the biological role of FABP in *F. hepatica* is associated with parasite survival via host immune response modulation^55^. It has been reported previously that intradermal injection of plasmid DNA carrying CsFABP gene (pcDNA3.1-CsFABP) into Sprague–Dawley (SD) rats induced a Th1-type immune response^56^.

Glutathione S-transferases (GSTs, “CRM22_011285_GST28”) are proteins of antioxidant glutathione conjugating system involved in phase II defense and detoxification of xenobiotics, endogenous toxins, and free radicals. They also play an important role in host-parasite interactions. In experiments on *F. hepatica* it was shown that these enzymes have prostaglandin-synthase activity^57^. Previously, we have shown that *O. felineus* GST has a high affinity for GST from related trematodes species^58^. A recent preclinical assessment of stem of *Nicotiana tabacum* on excision wound model demonstrated high levels of GST in wound area, which was associated with a successful wound healing process^59^.

In addition to that, protein Thioredoxin peroxidase (TPx, “CRM22_009463_ Thioredoxin peroxidase”) was isolated in big comparative amounts. It plays an important role in maintaining redox homeostasis and in protecting organisms from the accumulation of toxic reactive oxygen species. Recombinant *Schistosoma japonicum* SjTPx-3 protein was previously shown to play a role in modulating host immune responses and influencing Th1/Th2 immune response switching^60^. GST and TPx are responsible for the host–parasite interaction and potentially participate in three of four phases of wound healing^57,60^.

Previously it was shown that wound healing agents, which possess high antioxidant and antimicrobial activity can provide a basis for the wide spectrum of perspective therapies for problematic wounds^11^. This suggests that multiple variable proteins with antimicrobial and antioxidant activity in the major fraction of ESP and Lysate samples potentially intensify and improve the quality of wound healing.

Previously it has shown that *F. hepatica* E-S proteins bind human plasminogen in vitro. It was demonstrated that enolase is responsible for this binding, suggesting that it may function as a plasminogen receptor^61^. Enolase (“CRM22_007121_Enolase”), identified in *O. felineus* proteome, is closely related to *F. hepatica* enolase, suggesting their functional identity as plasminogen receptor as well as the participation in early phases of wound healing. It is noteworthy that no granulin protein was detected in *O. felineus* ESP and Lysate samples, which requires further studies and, probably, the comparison to *O. viverrini* protein expression pattern^62^.

Thus, a wide range of pathological conditions in which wound healing manifests as a long, poorly organized process dictates to search for new effective therapeutic approaches to stimulate healing and restore skin integrity. Currently, in the cutting edge of healthcare, the researchers and physicians are especially interested in drugs with active substances represented by bioactive peptides of various micro- and macroorganisms. At present moment, there are only a few works demonstrating wound healing potential of individual fractions or proteins of helminths and non-parasitic nematodes as wound healing agents. Therefore, we demonstrate for the first time that liver fluke *O. felineus* excretory-secretory product and lysate samples have wound healing properties. Further studies of ESP and lysate protein fractions seem to be extremely promising for searching new bioactive peptides to correct various pathological conditions that result in the formation of chronic non-healing wounds.

## Materials and methods

### Parasites, animals and experimental design

#### Metacercariae of *Opisthorchis felineus*

Metacercariae of *O. felineus* were collected from naturally infected *Leuciscus idus* from the Ob River, Novosibirsk city, Western Siberia, and isolated from muscle tissues through digestion with pepsin–HCl overnight at 37 °C. The fish were collected from neither conservation areas nor private property and were not otherwise protected; hence, fishing permits were not required*. Leuciscus idus* is not considered endangered or rare, and the fishing methods complied with the Federal Law N166-F3 of 20.12.2004 (ed. 18.07.2011) “Fishing and conservation of water bio-resources”^20^.

#### Obtaining *Opisthorchis felineus* adult worms

Syrian hamsters [n = 5, were obtained from Conventional Animal Facility of the Institute of Cytology and Genetics (Novosibirsk, Russia)] were infected with 100 metacercariae. After 6 months, animals were sacrificed via carbon dioxide (CO_2_) inhalation for 4 min. The worms were isolated from the gallbladder and hepatic bile ducts. Manual selection of viable worms was performed under binocular light microscope, and then worms were washed several times with sterile saline (0.9% NaCl).

#### Lysate from adult *O. felineus*

For obtaining the lysates: (1) adult worms (n = 100) were crushed with scissors into Petri dishes on ice; (2) 500 µl of Lysis buffer (50 mM Tris–HCl (pH = 8.0), 0.1% SDS, 150 mM NaCl, 0.5% TritonX100, 0.2 mM PMSF) was added per 5 mg of tissue; (3) tissue was homogenized with a plastic homogenizer; (4) lysate was sonicated (Vibra-Cell VCX130) on ice at 30% amplitude 2 times, for 10–20 s; (5) protease inhibitor cocktail was added (#M221-1ML, Amresco, USA); (6) total sample was incubated on a rotator in a refrigerator for 1.5–2 h; (7) centrifuged at 12,000 rpm for 20 min; (8) supernatant was aliquoted and stored at − 80 °C. Protein concentration was measured spectrophotometrically using a commercial BCA Protein Assay kit (ThermoScientific) in accordance with the manufacturer's recommendations using BioPhotometer Plus equipment (Eppendorf, Germany).

#### Excretory-secretory product from adult *O. felineus*

*Opisthorchis felineus* adult worms (n = 150) were kept in a 6-well plate at 37 °C with 5% CO_2_ for 24 h, in the medium: RPMI (Thermo Scientific, USA), 1% glucose, 100 µg/ml streptomycin (Sigma-Aldrich, USA), 100 IU/ml penicillin (Sigma-Aldrich, USA). After incubation, the medium was collected and the secretory product was isolated. Isolation steps: (1) to remove cellular and tissue debris, the medium was centrifuged at 4 °C, 300–500*g* for 10 min; (2) supernatant was centrifuged at 4 °C, 2000*g* for 20 min; (3) supernatant was centrifuged at 4000*g* for 10 min at 4 °C; (4) supernatant was filtered through a filter with a pore size of 0.22 μm (MF-Millipore, USA); (5) filtrate was loaded into a centrifuge concentrator (MWCO = 5 kDa), centrifuged at 4 °C, 6000*g* for 2 h; (6) for solution dialysis, sterile cold PBS (sodium phosphate buffer) was added to the supernatant, then placed in a centrifuge concentrator (MWCO = 5 kDa), centrifuged at 4 °C, 6000*g* for 30 min; (7) a cocktail of protease inhibitors (#80-6501-23, GE Healthcare, USA) was added to the concentrate; (8) concentrate was aliquoted, stored at − 80 °C. Half of the samples further undergone endotoxin removal using Pierce High Capacity Endotoxin Removal Resin according to the manufacturer’s instructions. Protein concentrations were measured spectrophotometrically using a commercial BCA Protein Assay kit (#23225, ThermoScientific, USA) according to the manufacturer's recommendations.

#### Wound healing in a mouse model

Two-month-old mice of the C57Bl/6 line (average weight 20–25 g) were obtained from Conventional Animal Facility of the Institute of Cytology and Genetics (Novosibirsk, Russia). All mice were individually housed in standard individually ventilated cages with unrestricted access to food and water.

Mice were anesthetized with isoflurane [2% isoflurane with oxygen (O_2_) at 1 L/min], hair on the back was shaved off, and superficial wounds with a diameter of 8 mm were applied using a stencil. Further, the animals were randomly divided into the 7 following groups (10 mice/group):

wounded without treatment (n = 10) (chlorhexidine (Chg));
vehicle (n = 10) (1,5% methylcellulose) (V);
nonspecific control (n = 10) (BSA 10 µg);
2. specific treatment (n = 40): ESP 10 µg (ESP), ESP without endotoxin 10 µg (ESPwe), lysate 10 µg (Lys 10), lysate 50 µg (Lys 50).

The experiment lasted 10 days. The animals were treated every 3 days of the experiment with simultaneous detection of the wound area.

In each group, the treatment of wounds was carried out according to the following scheme:

1. antiseptic treatment (chlorhexidine);
2. application of substances (for all groups, the test substance was placed in 1.5% methylcellulose diluted in PBS, except chlorhexidine);
3. application of a Luxplast liquid plaster-spray (Farmac-zabban, Italy).

All non-specific control groups were added to exclude individual effects on wound healing processes: antiseptic (chlorhexidine group), neutral gel (1.5% methylcellulose group) and protein fraction (BSA group). Methylcellulose was used to gel (gel-solidify) the treatment solution and prevent the solutions from spreading over the skin. The application of a liquid patch-spray is necessary to reduce the pain syndrome, the absence of contamination and the research interest of the animal (Table S1, Supplementary material 1). This protocol was standard and has been validated^23^. A vehicle group was chosen as a reference group (nonspecific control).

The protocol was created with minimization of stress and pain of animals. All the hamsters and mice were examined daily for signs of illness, injury, or abnormal behavior by Conventional Animal Facility trained personnel. Food and water availability and the macroenvironment (temperature, humidity, noise, light intensity, and cleanliness) were also evaluated daily. No unexpected deaths of animals were registered during this study.

**Figure 7:**
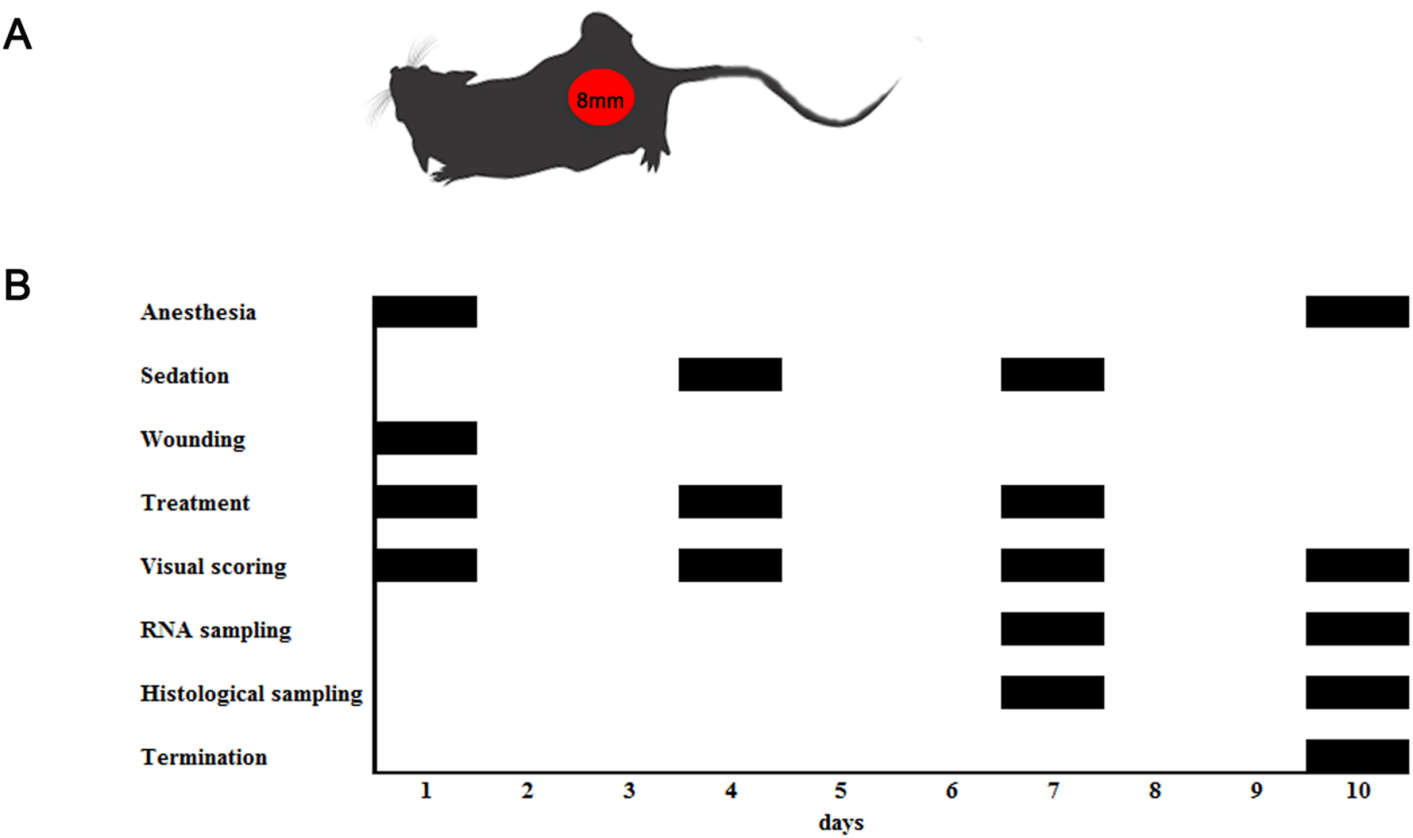
Schematic presentation of the experiment setup. (**A**) Each C57Bl/6 mouse (n = 70) was inflicted with superficial wound (8 mm diameter); (**B**) The entire duration of experiment was 10 days, during which the animals were anesthetized twice and received a sedation twice. All groups received treatment every 3 days of the experiment with simultaneous detection of the wound area. Animals were withdrawn from the experiment on days 7 and 10 of treatment with simultaneous collection of damaged skin samples for histological examination and for gene expression analysis.

Animals were withdrawn from the experiment on days 7 and 10 of treatment with simultaneous sampling (Fig. 7A,B).

4-stage blinding study protocol was applied: a first researcher divided the groups of animals based on randomization and was aware of what treatment the animals received; a second researcher administered anesthesia and monitored the animals; a third researcher performed all surgical interventions, selected material for research; a fourth researcher assessed wound areas, described morphological changes, analyzed gene expression.

#### Ethical statement

All the procedures were in compliance with EU Directive 2010/63/EU for animal experiments. Study design protocols and standard operating procedures (concerning the hamsters, the mice and the fish) were approved by the Committee on the Ethics of Animal Experiments at the ICG SB RAS (permit number 25 of 12.12.2014 (approved for the hamsters and the fishes); permit number 123 of 20.07.2022 (approved for the mice)). All methods were reported in accordance with ARRIVE guidelines (https://arriveguidelines.org).

#### Visual wound scoring

Every 3 days of the experiment (Fig. 7B), the animals were photographed on a special substrate with a measuring ruler. Further, in the photographs, the area of the wound was measured using the CorelDRAW software. Then the percentage of wound healing was determined for each mouse separately.

#### Sample collection

The wound tissue was divided into 2 parts. Part of tissue was fixed in 10% aqueous neutral formalin (Biovitrum, Russia), the other part of tissue was immediately placed in RNA-later solution and stored at − 20 °C for further RNA isolation (Fig. 1B).

##### Histopathological examination

Skin samples exposed to 10% neutral formalin were dehydrated in a graded series of ethanol and in xylene (STP-120, Thermo Scientific). Dehydrated samples were enclosed in a paraffin medium HISTOMIX (BioVitrum). For microscopic examination, sections of 3.5 μm thickness were prepared on a rotary microtome Microm HM 355S (Thermo Scientific).

The resulting paraffin sections were stained via a standard protocol with hematoxylin and eosin and Mallory staining (detecting connective tissue fibers). To determine the stage of wound healing, an immunohistochemical analysis was performed (immunohistochemical SpringBioScience kit HRP-125) using specific primary antibodies, to analyze:

1. state of the extracellular matrix: collagen I (Abcam, cat. # ab34710, 1:200), α-smooth muscle actin (α -SMA; Abcam, ab7817, 1:300);
2. neoangiogenesis: CD34 (Abcam, cat. # ab81289, 1:300);
3. excretory-secretory product and lysate proteins: GST, TPX^24,25^.

Staining was performed according to the manufacturer’s protocol. The visualization was carried out under an AxioImager A1 microscope (Zeiss, Germany) with camera AxioCam MRc (Zeiss, Germany). Semi-quantitative analysis on histological sections was performed to assess the presence of a wet crust ("+/−"), epithelial ridges ("+/−") and area of infiltration ("+++"—more than 60% of the wound area, "+ +"—from 20 to 60% of the wound area, "+"—less than 20% of the wound area). The percentage of infiltrate area was determined in mm2 using CorelDRAW (version number 22.0, https://www.corel.com/en/) and ImageJ (version number 1.50i, http://imagej.nih.gov/ij) software. Using a closed test system for 100 points (Morphometry and ImageJ software) the percentage of connective tissue and number of CD 34+ positive new formed blood vessels in the wound area was determined.

##### Gene expression analysis

Total RNA was isolated using the ExtractRNA (Evrogen, Russia). Concentrations of RNA were determined on a NanoDrop spectrophotometer (ND1000, NanoDrop Technologies, USA). First-strand cDNA synthesis was performed with the RevertAid Kit (Fermentas, European Union). Expression levels of the genes were measured by real-time PCR with the EVA Green Reagent Mix (Synthol, Russia) on a CFX96 real-time PCR system (Bio-Rad, USA). *Gapdh* and *Hprt1* genes were chosen as an endogenous internal control. Triplicate real-time PCRs were performed on each sample. The fold-change in target gene expression (normalized to the controls) was calculated from threshold cycle values (Ct; CFX96 software). Sequences for all primers and probes can be found in Table S2 (supplementary material 1) (Synthol, Russia).

### Proteomic analysis

The proteomic analysis was performed at the “Human proteome” Core Facility of the Institute of Biomedical Chemistry (Moscow, Russia).

Proteomic analysis approach called GeLC-MS/MS was chosen to study of the excretory-secretory product and lysate proteins. This approach is based on one-dimensional sodium dodecyl sulfate–polyacrylamide gel electrophoresis (SDS-PAGE), in-gel protein digestion with trypsin followed by liquid chromatography-tandem mass spectrometry. The SDS-PAGE step allow to removes hemozoin also as detergents, buffers and salts from the protein extract that may interfere with mass spectrometry analysis^63,64^.

Fast denaturing one-dimensional electrophoresis was carried out in the presence of SDS with a polyacrylamide concentration of 8 to 12% in a separating gel and 5% in a concentrating gel. A total of 25 μL of the sample was applied to the lane, and electrophoresis was carried out for 20 min at constant current (18 mA). In this case, the voltage was 50 V for the first 10 min and increased to 100 V from 11 to 20 min. The samples were fixed with a solution of acetic acid and ethanol (2 times for 20 min). The protein line (total long ~ 6 mm) was cut into three approximately equal fragments about 2 mm wide and about 10–12 mm long. The obtained gel fragments were washed 2 times for 15 min with 100 μL of 50 mM ammonium bicarbonate solution, then dried with 100% acetonitrile (15 min), after which the solution was removed, and the samples were dried on a centrifugal vacuum evaporator (Concentrator Plus, Eppendorf). Proteins in each gel fragment were distained and digested by trypsin using the protocol described previously^65,66^.

The LC–MS/MS of obtained peptides was perfomed by the method described below^65^.

Proteomic analysis of peptides was carried out using an Ultimate 3000 RSLCnano chromatographic HPLC system (Thermo Scientific, USA) connected to a mass spectrometer Q-exactive HFX (Thermo Scientific, USA). Peptides were separated with high-performance liquid chromatography (HPLC, Ultimate 3000 Nano LC System, Thermo Scientific, Rockwell, IL, USA) in a 15-cm long C18 column (Acclaim^®^ PepMap™ RSLC inner diameter of 75 μm, Thermo Fisher Scientific, Rockwell, IL, USA). The peptides were eluted with a gradient of buffer B (80% acetonitrile, 0.1% formic acid) at a flow rate of 0.3 μL/min. The total run time was 90 min.

MS analysis was performed in triplicate with a Q Exactive HF-X mass spectrometer (Q Exactive HF-X Hybrid Quadrupole-OrbitrapTM Mass spectrometer, Thermo Fisher Scientific, Rockwell, IL, USA). The temperature of capillary was 240 °C and the voltage at the emitter was 2.1 kV. Mass spectra were acquired at a resolution of 120,000 (MS) in a range of 300–1500 m/z. Tandem mass spectra of fragments were acquired at a resolution of 15,000 (MS/MS) in the range from 100 m/z to m/z value determined by a charge state of the precursor, but no more than 2000 m/z. The maximum integration time was 50 ms and 110 ms for precursor and fragment ions, correspondently. AGC target for precursor and fragment ions were set to 1 * 106 and 2 * 105, correspondently. An isolation intensity threshold of 50,000 counts was determined for precursor’s selection and up to top 20 precursors were chosen for fragmentation with high-energy collisional dissociation (HCD) at 29 NCE. Precursors with a charge state of 1+ and more than 5+ were rejected and all measured precursors were dynamically excluded from triggering of a subsequent MS/MS for 90 s^65^.

The obtained raw data were processed using the MaxQuant software (version 1.6.3.4) with the Andromeda search engine^67^.

The database of predicted protein sequences from *O. felineus* genome (GenBank: PRJNA413383) was used to identify proteins. The identification settings were as follows: trypsin as a specific protease with a maximum of 2 missed cleavage, a maximum m/z deviation of 5 ppm was allowed for precursor’s identification and 10 ppm were set as match tolerance for fragment identification (acquisition in Orbitrap), Oxidation of methionines, N-terminal protein acetylation and modification of cysteine with propionamide was set as variable modification for the peptide search. Peptide Spectrum Matches (PSMs), peptides and proteins were validated at a 1.0% false discovery rate (FDR) estimated using the decoy hit distribution. Proteins were considered as significantly identified if at least two peptides were found for them. Label-free protein quantification was based on iBAQ^65^.

### Statistical analysis

Statistical data analysis was performed using the Statistica 6.0 program (Statsoft, USA). The data were expressed as a percentage of the maximal possible score and presented as a heat map using the heatmap.2 (v.3.1.3) R package (https://www.rdocumentation.org/packages/gplots/versions/3.1.3/topics/heatmap.2). The non-parametric Kruskal–Wallis test was used to compare experimental groups with control groups (vehicle, unwounded healthy skin) or with previous study period; cluster analysis was used for proteomic analysis ESP and Lysate samples. Values of *^,#^p < 0.05; **^,##^p < 0.01; ***p < 0.005 were considered statistically significant.

## Supplementary Information

Supplementary Tables.

Supplementary Figure S1.

## Data Availability

The datasets generated and analyzed during the current study are available in the PRIDE repository, accession number PXD037991.
