# Author Correction: Wound healing approach based on excretory-secretory product and lysate of liver flukes

**DOI:** 10.1038/s41598-025-03488-5

**Published:** 2025-06-10

**Authors:** Anna V. Kovner, Alena A. Tarasenko, Oxana Zaparina, Olga V. Tikhonova, Maria Y. Pakharukova, Viatcheslav A. Mordvinov

**Affiliations:** 1 Institute of Cytology and Genetics, Siberian Branch of Russian Academy of Sciences (ICG SB RAS), 10 Lavrentiev Ave., Novosibirsk, 630090 Russia; 2 Department of Natural Sciences, Novosibirsk State University, 2 Pirogova Str., Novosibirsk, 630090 Russia; 3 Institute of Biomedical Chemistry (IBMC) RU, Pogodinskaya 10, Moscow, 119121 Russia

Correction to: Scientific Reports 10.1038/s41598-022-26275-y published online 13 March 2025.

The original version of the Article contained an error in Figure 5. During the figure assembly, the “10 days” panel image in Figure 5G was duplicated.

**Fig. 5:**
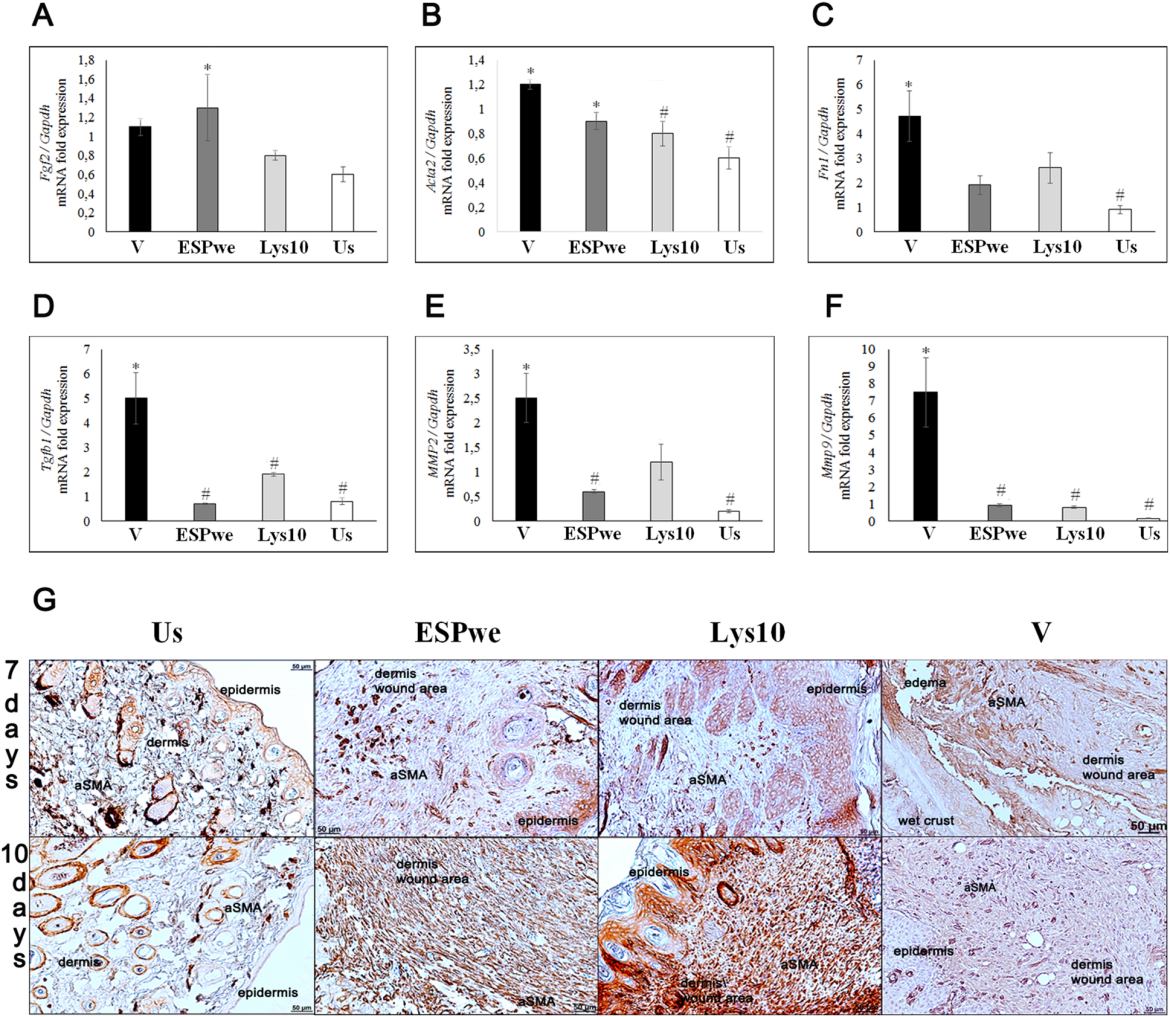
Skin extracellular matrix study. Specific treatment (ESPwe and Lysate 10 μg) significantly reduced expression of genes responsible for ECM remodeling. (A–F) *Fgf*2, *Acta*2, *Fn*1, *Tgfb*1, *Mmp*2, *Mmp*9 genes were normalized to average *Gapdh* expression. Data is presented as mean ± SEM, *p ≤ 0.05, **p < 0.01 as compared to unwounded healthy skin group, #p ≤ 0.05 as compared to a vehicle group; (g) α-SMA positive cells and tissue fibers in a wound area and in an un-wounded healthy skin.

The original Figure 5 and accompanying legend appear below.

The original Article has been corrected.

