# Supplementary material for: Wound healing approach based on excretory-secretory product and lysate of liver flukes
Source: Sci Rep. 2022 Dec 14;12:21639. doi: 10.1038/s41598-022-26275-y (PMC9751068; doi:10.1038/s41598-022-26275-y)
Supplement: Supplementary file 2 — Supplementary Figure S1. [file 41598_2022_26275_MOESM2_ESM.pdf]

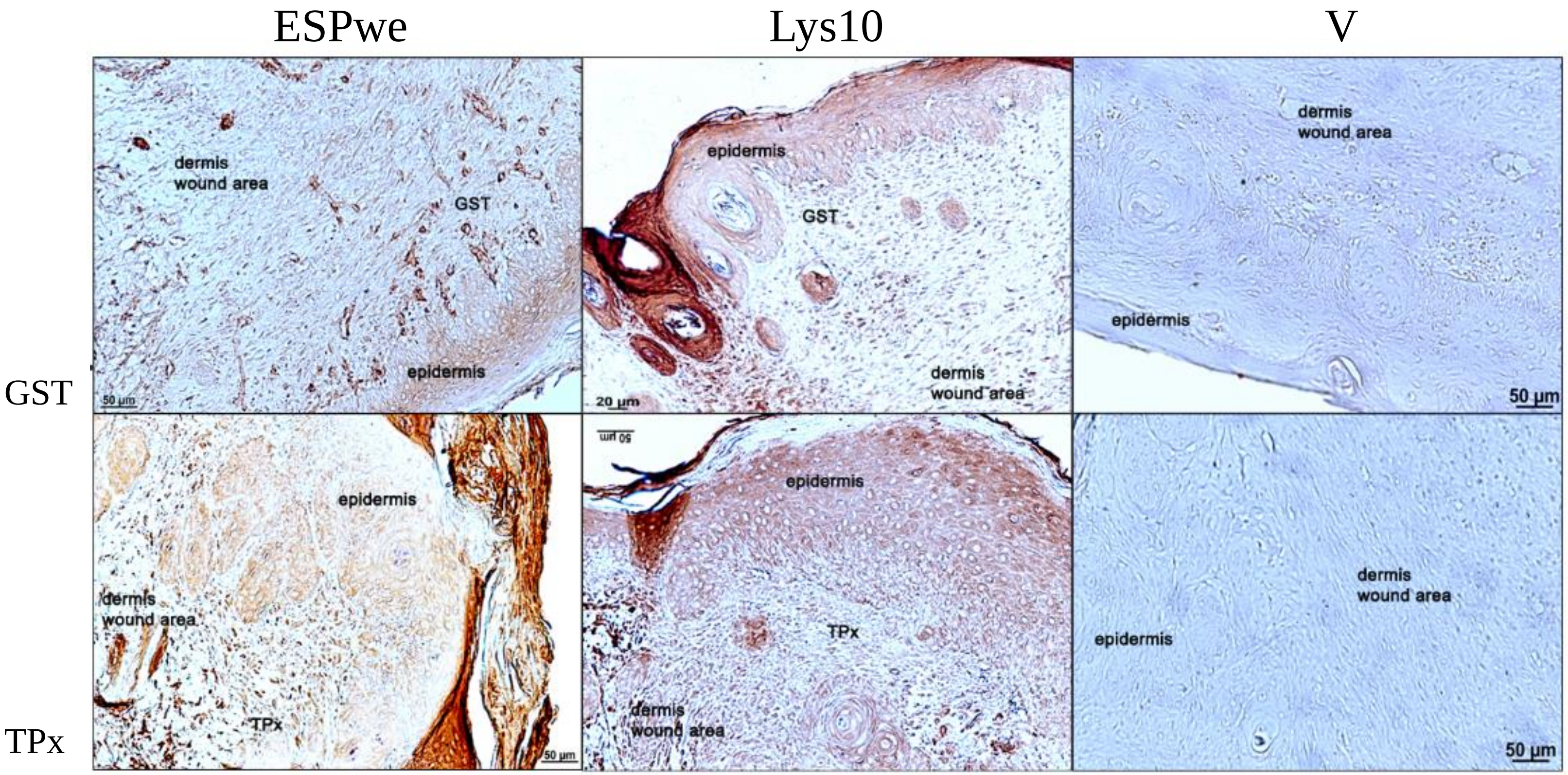

Figure S1. GST and TPx (major fraction proteins both ESP and Lysate samples) positive cells in the epidermis and dermis (wound area), 7 days after treatment, IHC staining. ESPwe – excretory-secretory product without endotoxin 10 µg, Lys10 – lysate 10 µg, V – vehicle.
